# Phytochemical, antimicrobial, antioxidant and enzyme inhibitory potential of medicinal plant *Dryopteris ramosa* (Hope) C. Chr.

**DOI:** 10.1186/s12906-021-03370-7

**Published:** 2021-07-08

**Authors:** Fiaz Alam, Syed Hurmat Ali Khan, Mohammad Hassham Hassan Bin Asad

**Affiliations:** 1 Department of Pharmacy, COMSATS University Islamabad, Abbottabad, Campus-22060 Pakistan; 2 Department of Genetics, Institute of Fundamental Medicine and Biology, Kazan Federal University, 420008 Kazan, Russia

**Keywords:** *D. ramosa*, Antibacterial, Antifungal, Cytotoxic, Antioxidant, Enzyme inhibition

## Abstract

**Background:**

*Dryopteris ramosa* has numerous potentials uses in the treatment of different maladies as old traditional medication**.** The fronds of *D. ramose* are edible and orally administered for producing antibiotic effect. They are also used as astringent and febrifuge, and as a pesticide.

**Methods:**

Extraction of fronds of *D. ramosa* using solvents of increasing polarity, namely, ethyl acetate, methanol and water were tested for phytochemical (qualitative tests, GC-MS), antimicrobial (well method), antioxidant (DPPH), antifungal (tube dilution), cytotoxic activity (brine shrimps lethality assay) and LOX and COX inhibitory activities were performed using standard methods.

**Results:**

The phytochemical analysis of the crude methanolic extract revealed that the fronds are rich in flavonoids, alkaloids, saponins, tannins, glycosides and triterpenoids. The total flavonoid content of the ethyl acetate fraction was 46.28 μg QE/mg extract. The GC-MS analysis revealed nine major compounds that constituted the crude drug and potentially had a role in reported activities. The crude extract was the most active amongst all the fractions against the bacterial and fungal strains used such that it inhibited the growth of *P. aeruginosa* with a zone of 13 mm and a MIC value of 16 μg/ml as compared to the standard cefixime, which inhibited the zone by only 10 mm and a MIC value of 32 μg/ml. The highest antioxidant potential in DPPH assay was shown by the crude extract with 91.948% free radical scavenging activity. The bring shrimps lethality potential of the crude extract was the highest, with a LD_50_ value of 47.635 μg/ml. The ethyl acetate fraction inhibits 91.36% of alpha glucosidase enzyme at a concentration of 0.5 mg/ml. In case of acetylcholine esterase inhibition assay, the methanol fraction inhibits 58.26% of the enzyme activity. Similarly, for butyrylcholine esterase inhibition, the maximum inhibitory effect was seen in the methanol fraction, with a percentage inhibition of 47.32%.

**Conclusion:**

These test results support traditional medicinal uses of the plant. *Dryopteris ramosa* could be imperative for being used as a therapeutic agent and the medicinal importance of this plant should be further investigated.

## Background

Pteridophytes (ferns and its allies) are reported to have medicinal uses and are locally used. The exploration and isolation of active constituents of which is yet to be done [1]. According to The National Aeronautics and Space Administration (NASA) and Korea Rural Development Administration (KRDA), ferns purifies the air from volatile organic compounds (VOCs), viz., *Nephrolepis obliterate* and *Nephrolepis exaltata*, commonly called Boston fern and Kimberly queen fern, respectively. There are around 12,000 recognized species of ferns worldwide and play a vital role as folk medicine. Among the 12,000 species, only 131 exist in Pakistan [2]. They are widely distributed in Himalayas [3, 4]. The ethnobotany of fern shows its importance as food, dye, bio-fertilizers, biogas production and medicinal source [5]. *Dryopteris ramosa* locally known as kunji or pakha is found in moist and shady places at high altitude. The fronds of *D. ramosa* are edible and orally administered for producing antibiotic as well as aphrodisiac effect. They are also used as astringent and febrifuge, as a pesticide. This study was designed to evaluate the medicinal plant *D. ramosa* for various phytochemical and biological activities to explore its potential use in traditional medicines.

## Methods

### Collection of plant material

The plant was collected from Galyat, Pakistan. Identified by a botanist, Dr. Manzoor Hussain and was placed in the herbarium at the botany department of Hazara University Mansehra with voucher specimen no. (BDHU-*D.R*-088/17). After identification, the plant was thoroughly washed with water and shade dried. Once completely dried, the plant was ground to fine powder. The powder was macerated in methanol (twice distilled) for 21 days, at room temperature such that the material was shaken and mixed twice a day. After the completion of 21 days period, the solvent was filtered from the macerated residue of the plant powder using muslin cloth, followed by filtration twice through filter paper. After filtration, the solvent was concentrated using rotary vacuum evaporator at 40 ° C. Once concentrated, it was allowed to be completely dried at room temperature and the weight of the crude methanolic extract was calculated. Different fractions of the crude extract were made by using liquid-liquid extraction with solvents in the increasing order of polarity such as ethyl acetate, methanol and water [6]. The crude extract was re-dissolved in distilled water and extracted with the solvents using a separating funnel. The crude fraction along with the solvent was shaken vigorously in the separating funnel and the layers were allowed to separate out. Once the layers were completely separated, the desired layer in the funnel was collected and concentrated. After concentration, each fraction was properly labelled, allowed to dry and stored in the refrigerator along with the crude fraction.

### Antibacterial assay

The antibacterial potential of the extracts was determined by agar well diffusion method by protocols already mentioned in literature [7]. Following organisms were used in the study: Methicillin resistant *Staphylococcus aureus*-10 (ATCC 33591), Methicillin resistant *Staphylococcus aureus*-11 (ATCC 29213), *Escherichia coli* (ATCC 15224), *Pseudomonas aeruginosa* (ATCC 9721) and *Staphylococcus aureus* (ATCC 6538). For the preparation of nutrient agar, 28 g of agar was dissolved in 1000 ml of distilled water. The media was autoclaved and poured into petri plates under sterile conditions. The plant extract samples were prepared by dissolving 20 mg of the extract in 1 ml of DMSO. Cefixime with the concentration as 1 mg/ ml of DMSO was used as the control whereas, DMSO was the negative control.

After the formation of bacterial lawns, wells were made into the agar using cork borer. Each well was sealed using 20 μL of the agar. After sealing, 40 μL of each extract were added in each well. The plates were then allowed to incubate at 37 ° C for 24 h. The experiments were run in triplicates. The zones of inhibition for extracts with good antimicrobial activity were determined in mm.

### Antifungal assay

The antifungal potential of the extracts was determined by agar well diffusion method by protocols already mentioned in literature on the selected strains of *Candida albicans* and *Candida kefyr* [7].

The plant extract samples were prepared by dissolving 20 mg of the extract in 1 ml of DMSO. Clotrimazole with the concentration as 1 mg/ ml of DMSO was the positive control while DMSO was the negative control. For the preparation of sabouraud dextrose agar, 65 g of agar was dissolved in 1000 ml of distilled water. The media was autoclaved and poured into petri plates under sterile conditions. After the formation of fungal lawns, wells were made into the agar using cork borer. Each well was sealed using 20 μL of the agar. After sealing, 40 μL of each extract were added in each well. The plates were then allowed to incubate at 37 ° C for 24 h. The experiments were run in triplicates. The zones of inhibition for extracts with good antifungal activity were determined in mm.

### Antioxidant assay

DPPH free radical scavenging assay was applied to assess the antioxidant capacity of the plant extracts by following protocols with little alteration [8]. For the plant extracts, 2 mg of each extract was mixed with 1 ml DMSO and placed on the vortex mixer for 10 min in order for proper mixing. For DPPH solution, 3.94 mg of DPPH was dissolved in 100 ml methanol hence 100 ml of 0.1 mM DPPH solution was acquired. One milliliter of the sample solution was mixed in 2 ml of 0.1 mM solution of DPPH and incubated for 30 min, in dark, at room temperature. After incubation, the absorbance was measured at 517 nm using UV spectrophotometer. The process was repeated three times and the percentage radical scavenging activity was determined as follows:

$$ \mathrm{Radical}\ \mathrm{scavenging}\%=\mathrm{Apc}-\mathrm{As}/\mathrm{Apc}\times 100\kern0.2em \left(\mathrm{Apc}=\mathrm{absorbance}\ \mathrm{of}\ \mathrm{the}\ \mathrm{positive}\ \mathrm{control};\mathrm{As}=\mathrm{absorbance}\ \mathrm{value}\ \mathrm{of}\ \mathrm{sample}\ \mathrm{solution}\right). $$

### Brine shrimp lethality assay

The cytotoxic assay of the plant extract was performed using brine shrimp lethality test with slight alteration [9]. Artificial sea water was made by dissolving 38 g of sea salt in 1 l of distilled water. The pH of this solution was maintained at 8.5. The plant extracts were made such that 5 mg of each extract was dissolved in 5 ml of methanol. A two chambered assembly that had a perforated wall between the two compartments was filled with artificial sea water. In on chamber, the brine shrimp eggs were placed and this compartment was covered with aluminum foil in order to retain darkness. The assembly was left for 48 h at 28 ° C ensuring no disturbance. To the other side of the compartment, a lamp was placed so that the hatched nauplii move towards the light and were collected. 5 mg/5 ml methanol solutions were made of each sample, of which 1000, 100 and 10 ppm dilutions were taken in test tubes in triplicate. After complete evaporation of methanol in each test tube, 10 nauplii along with 4.5 ml of sea water was taken and incubated for 24 h. Sufficient oxygen and humidity conditions were maintained in the incubator. After 24 h of incubation, number of dead nauplii were counted and the LD_50_ value of the extract was determined using probit analysis software [10].

### Phytochemical analysis

The phytochemical analysis of different fractions of the fronds was carried out in order to determine their qualitative and quantitative profile. The qualitative identification tests of the crude methanolic extract was carried out by using standard protocols with slight modification in the procedure [11, 12]. Total flavonoid content *were determined by using* the aluminum chloride calorimetric method, the total flavonoid content (TFC) of the extracts were determined by the previously described assays with slight modification [13].

### Acetylcholine esterase inhibition assay

The inhibition potential of the extracts against acetylcholine esterase enzyme was determined with slight modification in the protocols according to system availability [14].

The enzyme was dissolved in the phosphate buffer such that to make the concentration as 0.005 units per ml. The enzyme solution was then stored at − 80 ° C. the test samples were dissolved in DMSO to obtain 20 mM solution. The extracts were diluted using sodium phosphate buffer.

### Butyrylcholine esterase inhibition assay

The inhibition potential of the extracts against acetylcholine esterase enzyme was determined with slight modification in the protocols according to system availability [15]. The enzyme was dissolved in the phosphate buffer such that to make the concentration as 0.005 units per ml. The enzyme solution was then stored at − 80 ° C. the test samples were dissolved in DMSO to obtain 20 mM solution. The extracts were diluted using sodium phosphate buffer.

### Alpha glucosidase inhibition assay

The alpha glucosidase activity was carried out according to the previously employed protocol with some modifications [16]. DMSO was used as a solvent to dissolve the samples. The enzyme solution with following composition was prepared fresh; α -glucosidase (0.8 units/ml) in 50 mM phosphate buffer (pH 7), substrate pNP-G (0.7 mM) in phosphate buffer and 100 mM NaCl. The mixture was kept cool during the test. 20 μL of test solution and 80 μL of enzyme solution were incubated for 5 min at 37 °C. 1.9 mL of substrate solution was introduced to initiate the reaction and placed for incubation for further 15 min. To stop the reaction 0.5 M Tris solution (2 mL) was mixed. The pNP (p-nitrophenol) formed during the reaction showed absorbance at 400 nm. DMSO (20 μL) acted as a blank. The results were compared with standard drug Acarbose. The α-glucosidase inhibition in percent was calculated by = 100-(AB-AS)/AB Where AB = absorbance of blank, AS = absorbance of the sample.

### GC-MS analysis

The GC-MS study of the crude extract of the fronds was done by the protocols already available in literature with slight modification [17].

50 mg of *D. ramosa* was extracted with methanol and added few drops of KOH and centrifuged for 10 min. The supernatant was analyzed on GC-MS. The instrument used as Perkin-Elmer GC Claurus 500 equipped with Elite-1 fused silica capillary column. Ionization source used as 70 eV. Helium was used as a carrier gas. The flow rate was 1 mL/min. Injection volume was 0.5 μL.

Injection temperature was 250 °C and of that of Ion source was 280 °C. The program was set as: oven temperature was held for 2 min at 110 °C at rate of 20 °C/min to 200 °C and then then at 5 °C/min to 280 °C held for 5 min. The run time was 40 min. The compounds were identified using NIST library by comparing the mass spectrum fragmentation patterns.

## Results and discussions

### Phytochemicals in fronds

The phytochemical analysis of the crude extract showed that secondary metabolites such as alkaloids, saponins, tannins, flavonoids, carbohydrates, triterpenes, phenols and glycosides are found to constitute the phytochemical profile of the fronds Table 1. The qualitative phytochemical analysis of the crude methanolic extract of *Dryopteris ramosa* fronds revealed the presence of phytochemicals such as flavonoids, saponins, glycosides, triterpenoids and tannins etc. Our study is in correspondence to a previously conducted study in which a number of plants were analyzed for their phytochemical constituents. Other than this organoleptic study was also carried out which can be helpful in the detection of adulterations and can confirm the authenticity of the intact of powdered drug and drug product. The ethno-pharmacological use of *Dryopteris ramosa* reveals that its phytochemical constituents are responsible for the subsiding of various ailments [18].

**Table 1:** Qualitative phytochemical analysis of crude extract of *D. ramose*

Sr. No.	Phytochemical	Test	Observations	Inference
1	Saponins	Froth test	Foam formation	+
2	Flavonoids	Shinoda test	Red or pink color	+
		Alkaline reagent test	Yellow color	+
		AlCl_3_ test.	Yellow ppt	+
3	Alkaloids	Dragendroff reagent	Reddish brown ppt	+
		Mayer reagent	Creamy ppt	+
		Wagner reagent	Reddish brown ppt	+
4	Carbohydrate	Fehling’s test	Brick red ppt	+
5	Glycosides	Keller-Killiani test	Brown ring at interface	+
		Legal’s test	Pink to red color	+
		Borntrager’s test	Reddish pink color	+
6	Triterpenes	Salkowaski test	Reddish brown color	+
7	Phenols	FeCl_3_ test	Color change	+
8	Tannins	FeCl_3_ test	Black color	+
		Gelatin test	Green black	+

### Total flavonoid content

**Fig. 1:**
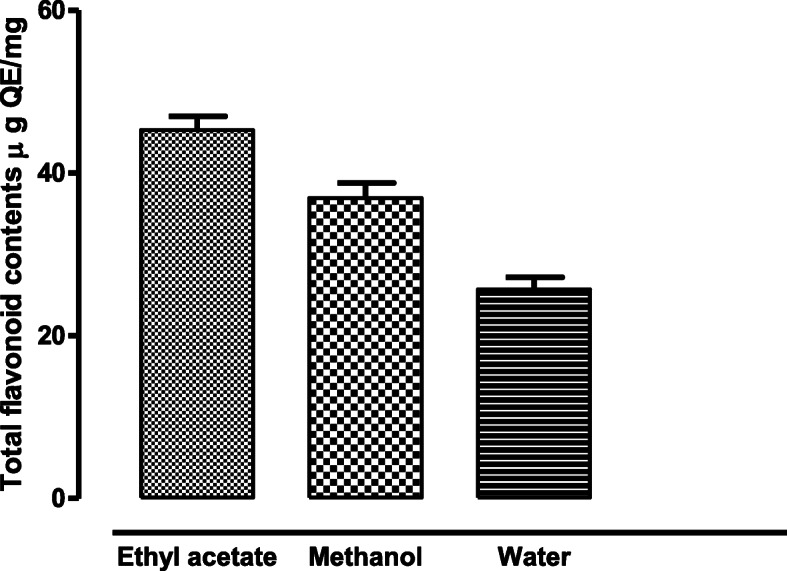
Total flavonoid contents in different fractions of *D. ramosa* extract

The total flavonoid content (TFC) of the different solvent fractions of crude extract was determined and their standard error mean was also determined. The results indicated that the ethyl acetate fraction showed the highest flavonoid content of 45.28 μg QE/mg of the extract with a standard error mean of ±1.67. Similarly, the methanolic fraction consists of 36.94 μg QE/mg of flavonoids with a standard error mean value of ±1.85. The water fraction, as compared to the other fractions showed the lowest value of TFC, with a value of 25.69 μgQE/mg with a standard error mean as ±1.52 (Fig. 1). The total flavonoid content (TFC) is an indicator of the quantitative measure of the flavonoids present in a sample. The total flavonoid content of the fronds showed that they are rich in flavonoids, especially the ethyl acetate fraction of the crude extract with a TFC value of 46.28 μg QE/mg of extract.

### α- glucosidase inhibition assay

**Fig. 2:**
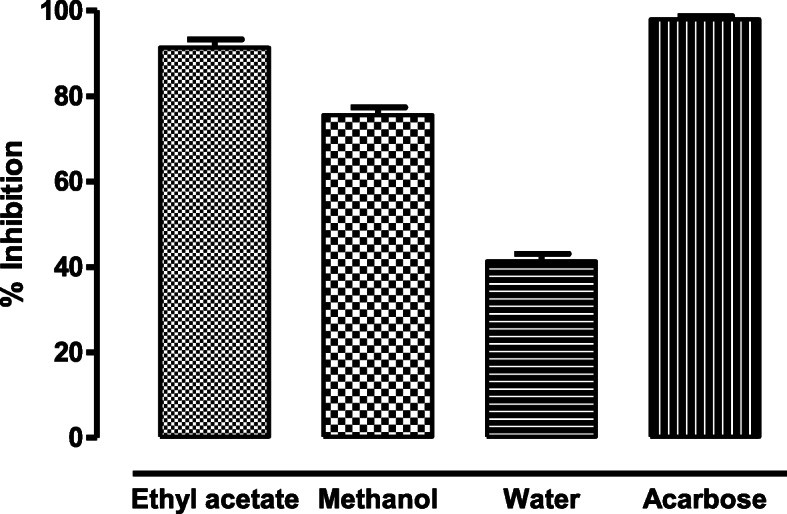
α- glucosidase inhibition activity of different fractions of *D. ramosa* extract

At a concentration of 0.5 mg/ml, the ethyl acetate fraction of the fronds inhibited 91.36% of the enzyme activity with a standard error mean of ±1.94. This was followed by the methanol fraction, in which the percentage inhibition was 75.54% (±1.83). The water fraction however inhibited only 41.35% (±1.75) of enzyme. The IC_50_ value calculated for the ethyl acetate fraction was 156.28 (±1.75) μg/ml. whereas methanol fraction showed IC_50_ value of 167.42 μg/ml (±1.59). Acarbose, as the standard showed IC_50_ value of 38.1 μg/ml (±1.25) (Fig. 2).

The major enzyme in the assimilation of sugars is alpha-glucosidase enzyme and its inhibitors restrict the postprandial hyperglycemia by impeding the freedom of D-glucose of oligosaccharides and disaccharides from dietary complex starches and in this manner postpone the glucose ingestion [19]. It is important to scan for successful and safe alpha-glucosidase inhibitors from natural sources, so as to create antidiabetic drugs. In our findings, the ethyl acetate fraction of the fronds inhibited 91.36% of the enzyme activity. This was followed by the methanolic fraction, in which the percentage inhibition was 75.54. However, the water fraction showed only 41.35% inhibition of the enzyme.

### Acetylcholine esterase inhibition assay

**Fig. 3:**
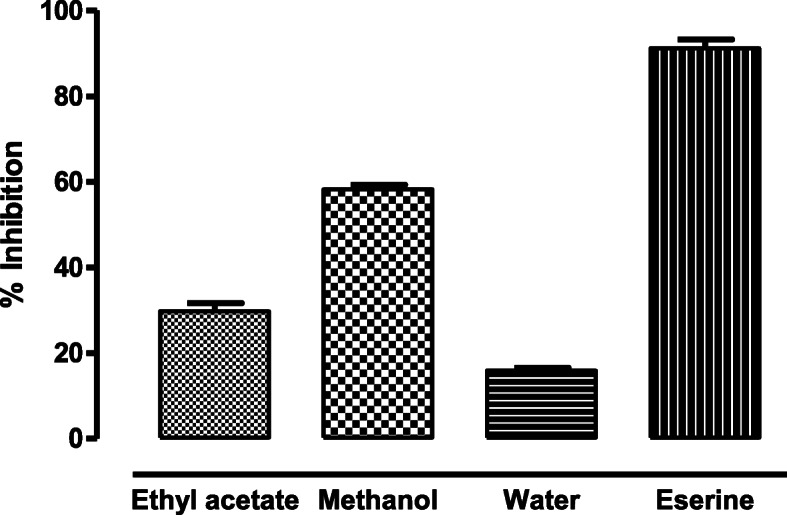
AChE inhibitory activity in different plant fractions of *D. ramosa* extract

The methanol fraction exhibited the best acetylcholine esterase inhibition activity, by inhibiting 58.26 (±1.95) % of the enzyme at a concentration of 0.5 mg/ml. The percentage inhibition recorded for the ethyl acetate and water fractions was 29.75 (±1.05) % and 15.92 (±0.65) % respectively. Eserine was used as a standard that inhibited 91.27(±1.99) % of the enzyme activity. IC_50_ value calculated for the methanolic fraction was 349.82 μg/ml whereas, for eserine it was 0.04 μg/ml (Fig. 3).

### Butyrylcholine esterase inhibition assay

**Fig. 4:**
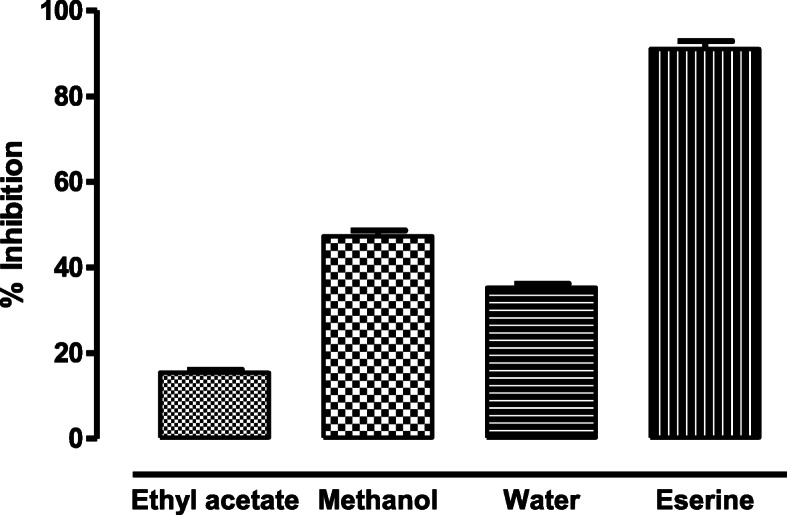
Percent inhibition of butyryl cholinesterase activity present in different fractions of *D. ramosa* extract

None of the fractions inhibited butyrylcholine esterase enzyme with a significant value. The inhibition shown by the extracts at a concentration of 0.5 mg/ml was 47.32 (±1.3) μg/ml, 35.26 (±0.95) μg/ml and 15.45 (±0.60) μg/ml for methanolic, water and ethyl acetate fraction respectively (Fig. 4). Acetylcholinesterase (AChE) and butyryl cholinesterase (BChE) are an important group of enzymes that are involved in the termination of synaptic transmission. The activity was performed against various fractions of the plant. At a concentration of 0.5 mg/ml, the methanol fraction of fronds exhibited the best acetylcholine esterase inhibition activity. However, none of the extracts inhibited the BChE significantly.

### Antibacterial assay

**Fig. 5:**
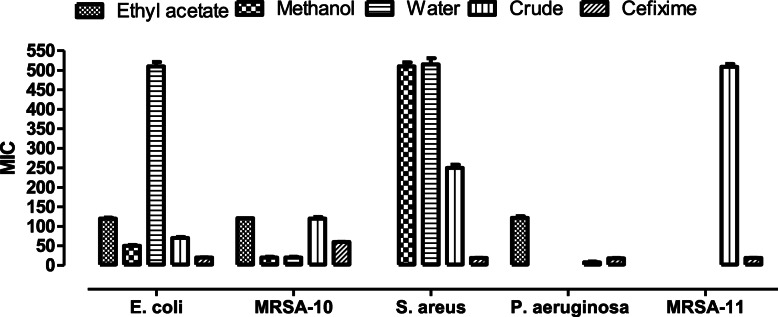
A report of MIC (antibacterial activity) against various pathogen posed by various fractions of *D. ramosa* extract

In case of antibacterial assay, different fractions were active against different strains and the zones of inhibition varied. The ethyl acetate fraction showed no activity against any of the bacterial strains. In case of the methanol fraction, a significant zone of 12 mm was produced against MRSA-10 strain, compared to the standard cefixime, in which the zone was only 10 mm. Similarly, against *E. coli*, the zone produced by the methanolic fraction was 8 mm. The water fraction produced a 12 mm inhibition zone against MRSA-10, as compared to 10 mm given by the standard and 6 mm zone against *S. aureus*. The crude extract was the most active, amongst all the fractions, against *P. aeruginosa*, where it exhibited a zone of 13 mm while the standard produced a zone of 11 mm. DMSO being the negative control did not inhibit the zones. The MIC was calculated for all the extracts. According to the results, the lowest MIC was shown by the crude extract against *P. aeruginosa*, with a MIC value of 16 μg/ml, as compared to the standard cefixime, which gave a MIC of 32 μg/ml. Similarly, against MRSA-10, the methanolic and water fraction have a MIC of 32 μg/ml, as compared to the MIC of cefixime that was 64 μg/ml (Fig. 5).

### Antifungal assay

**Fig. 6:**
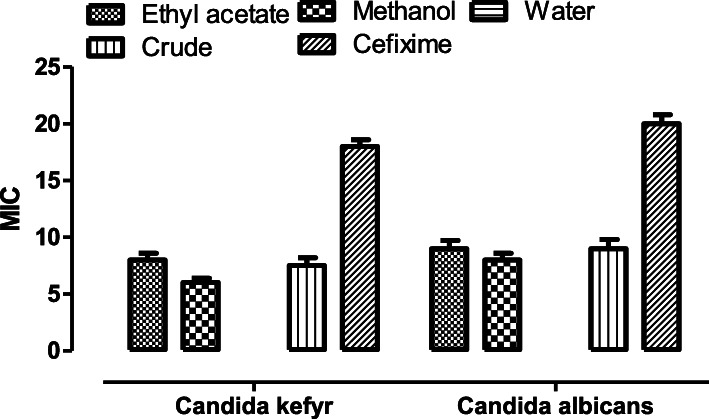
A report of antifungal activity against various pathogen showed by various fractions of plant extract

As compared to the antibacterial results, the antifungal results exhibited by the extracts were insignificant. Clotrimazole as standard inhibited the two fungal strains, *C. albicans* and *C. kefyr*, by producing an inhibiting zone of 20 mm (±0.8) and 18 mm (±0.6) respectively (Fig. 6). The antifungal activity exhibited by all the fractions, as well as the crude extract were insignificant, however, the antibacterial potential of the extracts was comparatively significant.

### Antioxidant assay

**Fig. 7:**
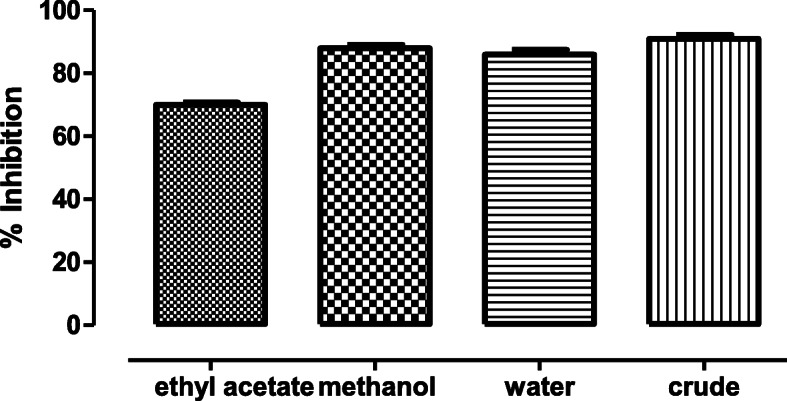
DPPH assay to map out antioxidant activity present in various fractions of active plant extract

According to the DPPH assay, the crude extract exhibited the highest free radical scavenging potential of 91.948% (±1.3), followed by the methanol fraction with the inhibitory percentage of 88.25% (±1.1). The water and ethyl acetate fractions had the percentage inhibition potential of 87.283% (±1.5) and 69.974% (±0.83) respectively (Fig. 7). Flavonoids and phenols are medicinally essential and are available as significant constituents in plants. They are generally viewed as in charge of antioxidant potential and in this way assessed for the most part for this reason. There are numerous reports that antioxidant potentials of plants are because of the nearness of phenolic compounds [20, 21]. The antioxidant potential of *Dryopteris ramosa* was determined using DPPH assay. According to the results, the crude extract exhibited the highest value of free radical scavenging potential, followed by the methanol fraction, water and ethyl acetate fraction.

### Brine shrimp lethality assay

For the lethality assay, the number of nauplii killed by the extracts were determined in order to calculate the LD_50_ values for the extracts. Methanol fraction, with a LD_50_ value of 47.63 μg/ml was determined to be the most toxic fraction amongst all. Whereas, the water fraction was comparatively the least toxic, with a LD_50_ value of 422.61 μg/ml. The order of lethality was water < ethyl acetate < crude extract < methanol. For the determination of the lethality of the extracts, brine shrimps’ assay was performed. The methanol fraction was found to be the most lethal fraction, whereas the water fraction was the least toxic fraction, as indicated by the brine shrimp assay.

### GC-MS analysis

The phytochemical constituents of the crude extract of *Dryopteris ramosa* were identified by using gas chromatography-mass spectrometry (GC-MS). The results of our GC-MS analysis indicated the presence of 9 compounds; 1-decene, 9-Methyl; Decane; 2-Heptane isothiocyanate; Heptyl octacosyl ether; Trichloroacetic acid, hexadecyl ester; Nonadecane, 1-chloro; Distearyl sulphide; 2-Methyltetracosan and 3-Butyl-2-nitrohept-1-ene. The identified compounds were found to be active pharmacologically. The isothiocyanate compounds has shown promising cholinesterase inhibitory activity previously [22]. Another compound identified by GC-MS was heptyl octacosyl ether which was reported to have strong antioxidant activity [23]. Similarly, trichloro acetic acid has application as safe agent for acne scars by its cytotoxic action [24]. Hexadecyl ester like compounds are reported to have a role as antimicrobial agent against uncomplicated UTI infection but the studies were not conclusive [25]. Nonadecane and related compounds reported to be the part of extracts previously tested for antioxidant and antimicrobial activity [26]. Distearyl sulphide and related compounds isolated from mangrove are reported to have bactericidal, insecticidal and fungicidal activities [27]. A complete description of the components obtained from GC-MS analysis was summarized in Table 2.

**Table 2:** GC-MS analysis of extract of *Dryopteris ramosa* with retention time of different compounds

Retention time (min)	Compound name	Molecular formula	Molecular weight
6.020	1-decene, 9-Methyl	C_11_H_22_	154
6.190	Decane	C_10_H_22_	142
7.060	2-Heptane isothiocyanate	C_8_H_15_NS	157
21.171	Heptyl octacosyl ether	C_35_H_72_O	508
22.162	Trichloroacetic acid, hexadecyl ester	C_18_H_33_O_2_Cl_3_	386
22.962	Nonadecane, 1-chloro	C_19_H_39_Cl	302
26.708	Distearyl sulphide	C_36_H_74_S	538
32.101	2-Methyltetracosan	C_25_H_52_	352
32.741	3-Butyl-2-nitrohept-1-ene	C_11_H_21_O_2_N	199

## Conclusions

*Dryopteris ramosa* has numerous potentials uses that are consolidated in the treatment of different maladies as old traditional medication. The results distinctly demonstrated that the ethyl acetate fraction of the fronds possesses the highest TPC value. The methanol fraction inhibits the alpha glucosidase enzyme such that the percentage inhibition can be regarded as a good activity. Similarly, the methanol fraction inhibits the AChE by marginally good percentage. *Dryopteris ramosa* crude extract demonstrated significant outcomes in the repression of cholinesterase enzyme. The crude extract showed the highest activity against *P. aeruginosa* so it could prove as a significant alternative in the treatment and prevention of diseases like meningitis, pneumonia etc. The antifungal potential of the plant is significantly low. The crude extract exhibits the highest antioxidant potential. Despite of all the activities given by the methanol fraction, it is the most lethal fraction, as revealed by the cytotoxic assay. As our results of investigation of *Dryopteris ramosa* uncovered the presence of large scale of significant elements in GC-MS analysis, this plant could be imperative for being used as a therapeutic agent. Therefore, the medicinal importance of this plant should be further investigated.

## Data Availability

The datasets used and/or analysed during the current study are available from the corresponding author on reasonable request.

## Abbreviations

*D. ramosa*: Dryopteris ramosa
GC-MS: Gas chromatography mass spectrometery
DPPH: 2,2-Diphenyl-1-picrylhydrazyl
MIC: Minimum inhibitory concentration
BChE: Butyrylcholinesterase enzyme
AChE: Acetylcholine esterase enzyme
TFC: Total flavonoid contents
pNP: P-nitrophenol
DMSO: Dimethyl sulphoxide
